# Case Report: Management of an Elderly Patient With Metastatic Radioiodine-Resistant Differentiated Thyroid Cancer in a Rural Community, Remote From Specialist Oncology Services

**DOI:** 10.3389/fendo.2020.581014

**Published:** 2021-02-01

**Authors:** Rachael Wybrew, Michael Loynd, Maria Wybrew, Leslie Samuel

**Affiliations:** ^1^ Prince’s Street Practice, Thurso, United Kingdom; ^2^ Cancer Nursing Service, Caithness General Hospital, Wick, United Kingdom; ^3^ Anchor Unit – Clinic D, Aberdeen Royal Infirmary, Aberdeen, United Kingdom

**Keywords:** remote management, lenvatinib, radioiodine-resistant, case report, metastatic thyroid cancer

## Abstract

This case report describes an elderly patient with radioiodine-resistant differentiated thyroid cancer and additional multiple metastases living in a rural setting, remote from the specialist oncology service. This case is of interest because effective systemic therapies for treatment-resistant cancers, such as lenvatinib, are now available but can potentially cause significant toxicities that require extensive medical management. Here, we discuss how patient care was provided collaboratively by the local community teams integrated with remote specialist oncology services. A 77-year-old patient presented with symptoms of cauda equina secondary to a large metastatic sacral deposit. The deposit was biopsied, and histology revealed a diagnosis of differentiated follicular thyroid cancer that was treated with external beam radiotherapy and thyroidectomy, followed by radioiodine. However, the disease was found to be resistant to radioiodine therapy, and the patient subsequently developed back pain due to new bone metastases. After further palliative external beam radiotherapy, the patient was started on systemic treatment with lenvatinib. Treatment has continued for more than 2.5 years with a slow but steady improvement in symptoms and quality of life. Monitoring and assessment of lenvatinib therapy and management of associated toxicities was coordinated remotely from a specialist cancer center over 200 miles away, using the skills of the local medical and nursing teams. This case report demonstrates how a cooperative effort using local teams and video-conferencing links to a specialist cancer center can be applied to safely treat a patient with a medication that may result in significant potential toxicities that require attentive and dynamic management.

## Introduction

Within the UK National Health Service, the oncological and palliative care service system in the Highlands and Islands of Scotland faces significant challenges due to the relatively sparse population spread over a huge geographical area, and where recruitment and retention of staff can be difficult (1). Here, with patients expected to travel over greater distances to access secondary healthcare, and with a limited set of available services compared with the rest of the UK (2), much of the day-to-day management of malignancy is coordinated locally through primary care services.

## Case Description

This case study provides details of a 77-year-old patient who presented with features of cauda equina, and who had subsequent diagnosis and management of metastatic differentiated-type follicular thyroid cancer coordinated from tertiary care centers more than 200 miles away from her home. Thus, this patient’s case provides an interesting discussion of patient-centered care within a challenging, resource-scarce, environment. Further, this patient’s treatment with lenvatinib, which shows promise for patients with radioiodine-resistant differentiated thyroid cancer, has been safely managed through coordinated care from local and specialist teams located a great distance away. This case indicates that this rare cancer can be treated remotely and safely with an agent with known toxicities and adverse events that require close monitoring, without requiring extensive travel by patients to clinics. However, patients should be assessed on a case-by-case basis to determine the feasibility of remote treatment.

**Figure 1:**
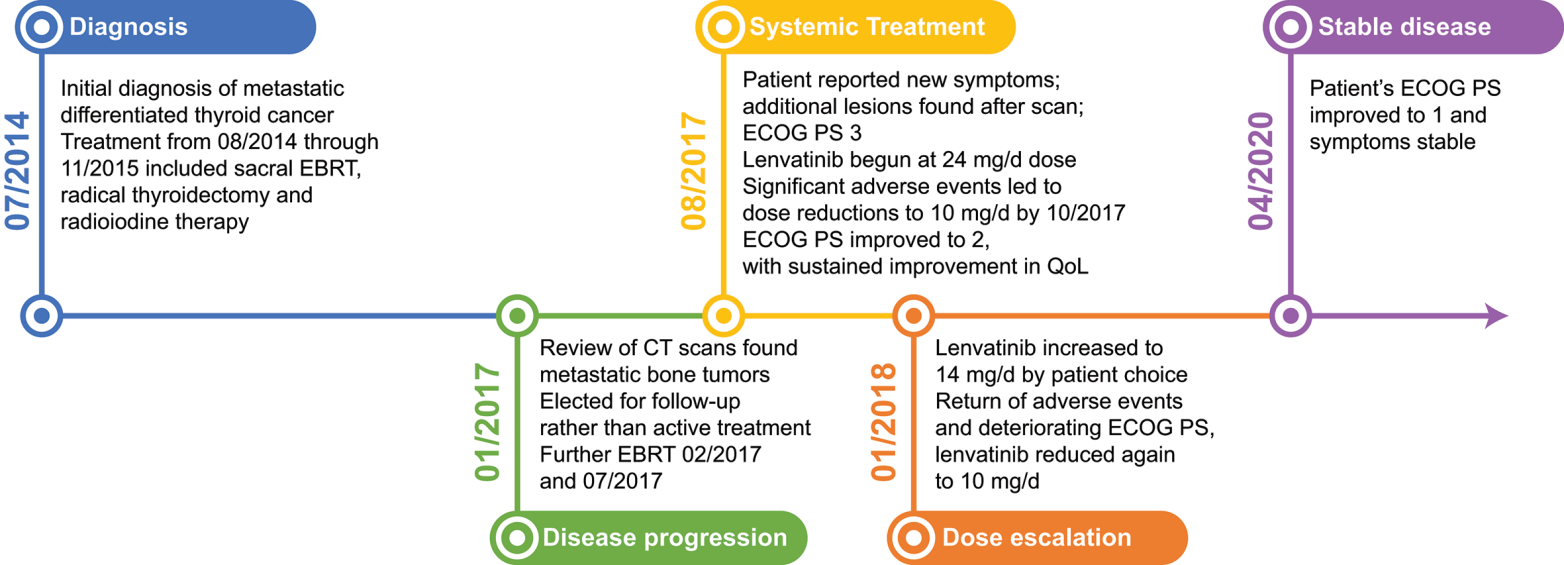
Case Report Timeline: Patient was initially diagnosed in July 2014, and received EBRT, a radical thyroidectomy, and radioiodine therapy. Metastatic bone tumors were found in January 2017 and the patient received additional EBRT. Seven months later the patient reported decreased quality of life and new symptoms and began systemic treatment with lenvatinib. Adverse events and dose modifications were managed cooperatively between the local treatment team and the specialist oncologist. As of April 2020, the patient remained stable, with an ECOG PS of 1. CT, computed tomography; EBRT, external beam radiotherapy; ECOG PS, Eastern Cooperative Oncology Group performance status; QoL, quality of life.

At a follow-up appointment following an elective knee-joint replacement in 2014, the patient presented with both urinary and occasional fecal incontinence with associated back pain (**Figure 1**). She had also experienced pain in her legs and was concerned whether this was a complication of the recent surgery. She reported depressed mood, poor sleep, loss of appetite, and constant exhaustion. Past medical history included a bowel obstruction, an inguinal hernia, hypertension (treated with perindopril since mid-2012), and mitral regurgitation.

## Diagnostic Assessment

**Figure 2:**
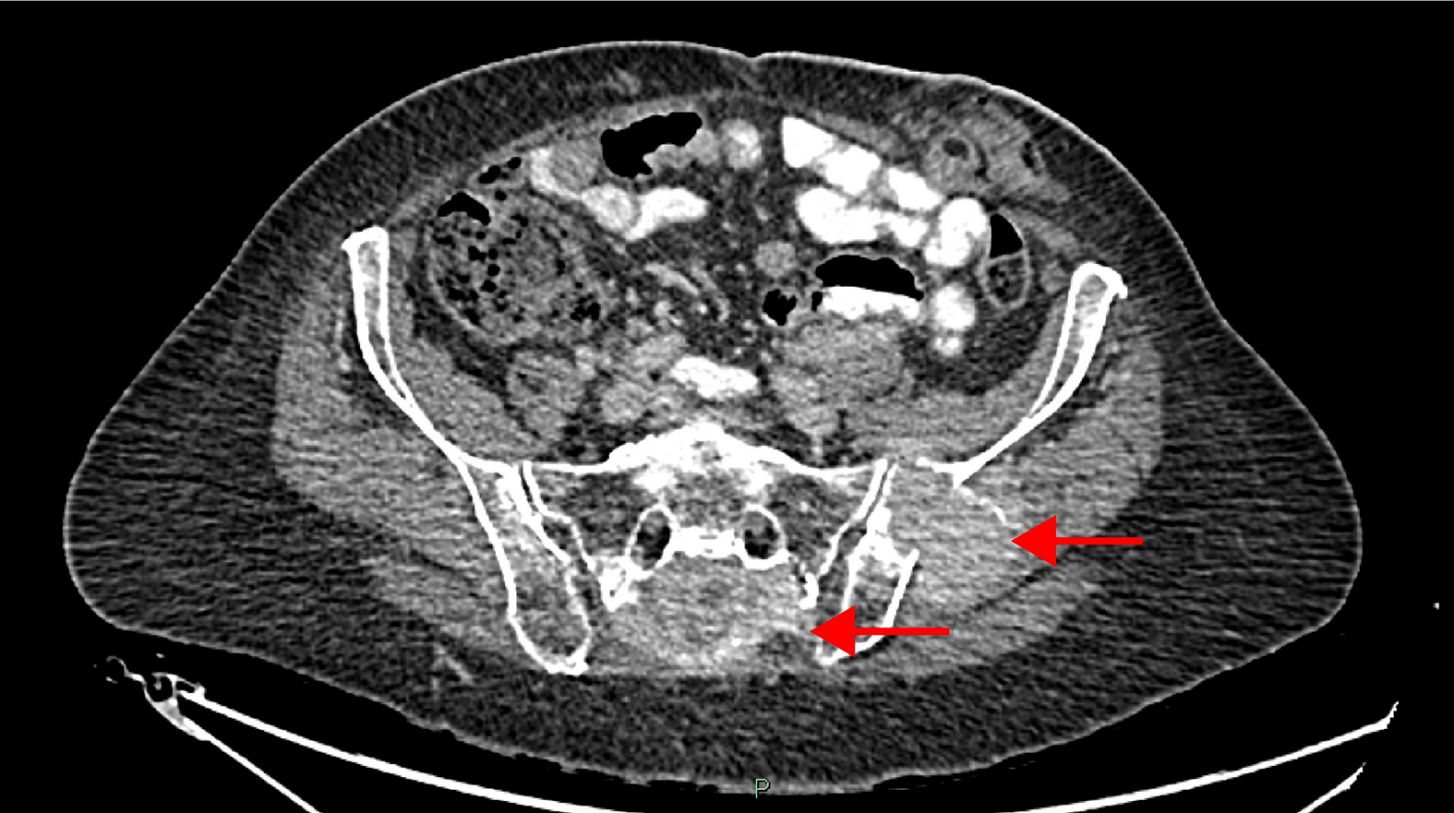
Sacral Mass at Diagnosis (July 2014).

The patient was admitted to a regional hospital in Inverness, more than 100 miles away, for an X-ray and magnetic resonance imaging scan of the spine, which showed a sacral mass of 9.0 cm x 4.7 cm x 4.0 cm encroaching on the S1–5 foramina as the likely cause of symptoms (**Figure 2**). A computed tomography (CT) scan of the chest, abdomen, and pelvis also showed an enlarged thyroid gland. A biopsy of the sacral mass led to a histological diagnosis of differentiated-type follicular thyroid tumor, originating in a 4-cm mass in the right lobe of the thyroid gland. After fine-needle aspiration biopsy, the mass was graded THY4 (suspicious lesion).

Approximately 1 month following confirmation of the diagnosis, the patient received external beam radiotherapy (EBRT) (20 Gy in 4 fractions to the sacral area) for symptomatic relief at a regional hospital in Inverness. The case was discussed by the North of Scotland thyroid cancer multidisciplinary team, who recommended the standard treatment of thyroidectomy with subsequent radioiodine therapy. As there was significant retrosternal extension of the thyroid gland, the patient was transferred to the specialist thyroid cancer team based in the university hospital in Aberdeen for a radical thyroidectomy, as surgery required both the thoracic and endocrine surgical teams to resect the thyroid gland. Pathology revealed a widely invasive follicular carcinoma, with vascular and capsular invasion.

On recovery from surgery the patient had a course of radioiodine treatment (3,750 MBq) in the university hospital in Aberdeen that had a specialist thyroid cancer team. Iodine uptake scanning following radioiodine treatment suggested new focal uptake in the costochondral junction of the second left rib, maxilla, mandible, soft tissue around the left lateral border of the hyoid, and left anterior pelvis, in addition to the known lesion in the sacrum. There was also some uptake of radioiodine in the thyroid bed, which may have been due to residual thyroid tissue. Failure to shrink the sacral mass meant that the patient continued to experience complete urinary incontinence. The patient had a urethral catheter sited, which gave her the confidence to leave the house and improved her quality of life; her Eastern Cooperative Oncology Group (ECOG) performance status (PS) was 1 at this time.

Over the following year, the patient received a further 3 rounds of radioiodine therapy in Aberdeen (5,420 MBq in March 2015; 5,710 MBq in July 2015; and 3,690 MBq in November 2015), for a total radioiodine dose of 18.57 GBq. On follow-up assessments through January 2016, the patient’s thyroglobulin (Tg) levels decreased from 90,400 to 9,400 ng/ml, but there was no reduction in the size of any of the tumor deposits (**Table 1**). Between the third and fourth round of radioiodine, the patient underwent colostomy surgery in the regional hospital in Inverness because of ongoing fecal incontinence. The urinary catheter and stoma resulted in significant improvements in the patient’s physical and emotional wellbeing, allowing her to perform activities of daily living unaided (with an ECOG PS of 1).

**Table 1:** Patient Characteristics Over Time.

Date	Thyroglobulin (ng/ml)	Thyroid-Stimulating Hormone (mU/L)^a^	Weight (kg)	Significant Events
Nov 2014	90,400	0.1	–	Post-thyroidectomy
Jan 2015	67,500	6.64	–	Post 1st RAI
Apr 2015	25,004	2.04	71	Post 2nd RAI
Jan 2016	9,400	0.65	–	Post 4th RAI
Jun 2016	7,330	0.06	–	–
Oct 2016	8,071	0.09	–	–
Feb 2017	5,802	0.02	–	PD - palliative radiotherapy
May 2017	3,470	0.04	–	–
July 2017	–	–	–	PD - further radiotherapy
Aug 2017	119,200	0.02	–	Lenvatinib started
Sep 2017	132,300	0.13	–	–
Nov 2017	10,493	0.43	66	–
Jan 2018	6,660	0.53	67	–
Oct 2018	10,451	11.27	67	–
Jan 2019	7,328	0.12	69	–
Mar 2019	8,246	0.08	68	–
May 2019	11,537	0.07	68	–
Jul 2019	16,372	0.11	69	–
Feb 2020	20,327	3.75	76	–

^a^The normal range for thyroid-stimulating hormone is 0.34–5.4 mU/L.

RAI, radioactive iodine treatment; PD, progressive disease.

Despite the appearance of metastases on imaging, the patient’s mobility and clinical condition significantly improved in the months following the final round of radioiodine, in keeping with the improving Tg results. The patient was prescribed levothyroxine to suppress thyroid-stimulating hormone, and Tg levels decreased to 7,330 ng/ml between November 2015 and June 2016 (**Table 1**).

The patient’s symptoms, imaging information, and blood test results were regularly reviewed remotely by the regional North of Scotland thyroid cancer multidisciplinary team. At a review of the patient’s bone scans in January 2017, metastatic disease progression was clinically determined and a trial period of lenvatinib treatment was suggested by the multidisciplinary team. The disease was suspected to be resistant to radioiodine because of the relapse after a substantial total dose of radioiodine that had been given in the prior year. A video-link consultation was coordinated by a cancer specialist nurse between the specialist oncologist, based in Aberdeen, and the patient, along with a supportive friend. As the patient’s condition was stable and her ECOG PS was still 1, it was agreed that continued observation rather than additional treatment with lenvatinib was appropriate at that time, given the possibility for adverse events from a systemic treatment to impair quality of life for an elderly patient.

In February 2017, the patient had further EBRT (8 Gy in one fraction, covering the T8–T10 area) at the regional hospital in Inverness. There was a good response to the EBRT, with the patient remaining reasonably active and her Tg decreasing to 3,470 ng/ml by May 2017 (**Table 1**). In the summer of 2017, the patient reported new symptoms of upper back and shoulder pain, and CT imaging at the local hospital revealed new tumor deposits in the thoracic spine, causing a compressive radiculopathy, with further tumor deposits in the left ilium. In keeping with this clinical progression, her Tg had significantly increased to 119,200 ng/ml (**Table 1**). The patient received additional radiotherapy (8 Gy in 1 fraction, covering the T3–T5 area) in July 2017 at the regional hospital in Inverness and was discharged with a reducing course of dexamethasone (starting at 16 mg/day). Despite this treatment, the patient experienced a reduction in mobility, with balance problems and difficulty walking, as well as reduced sensation and power in her legs and feet. She became immobile without a wheelchair and required social support at home. At this point, the patient’s ECOG PS was 3. The dose of dexamethasone was increased, due to symptomatic burden, from a tapered-down dose of 4 mg/day back to 8 mg/day. At a subsequent video-link consultation with the specialist oncologist—based on the patient’s reduced mobility, worsening ECOG PS, increased Tg levels, and discovery of new tumor lesions—it was agreed that the patient should start lenvatinib therapy.

Lenvatinib treatment began in August 2017 at a dose of 24 mg/day and the patient, together with a close friend and the local cancer nurse specialist from a local hospital, joined a video-link consultation every 2 weeks with the specialist oncologist in Aberdeen. This planned close/frequent review allowed for the detection and management of any significant adverse events with lenvatinib treatment, and for appropriate dose alterations of lenvatinib. Monitoring included blood tests, weight, ECOG PS, and regular self-monitoring of blood pressure.

Initially lenvatinib was well tolerated, with a grade 1 skin rash and an increase in blood pressure that was treated with amlodipine. However, by the end of cycle 1, the patient had significant thrombocytopenia: her platelet count fell to 129/µL, which was considered to be related to lenvatinib treatment and the previous radiotherapy to the sacrum and thoracic spine, both areas of significant bone marrow production. The amlodipine dose was increased to 10 mg/day. Lenvatinib treatment was paused for 2 weeks until platelet levels recovered and was then restarted at a lower dose of 20 mg/day. However, the patient’s platelet count decreased again, prompting a further stepped-down dose reduction to lenvatinib 10 mg/day in October 2017.

In November, after 3 months of lenvatinib therapy, a follow-up CT scan was performed and reviewed at the local hospital and found stable disease with no evidence of new tumor growth. The patient’s Tg levels had initially increased from 119,200 ng/ml at the start of the lenvatinib treatment to 132,300 ng/ml in September 2017, but then declined to 10,493 ng/ml in November 2017 (**Table 1**). As the patient’s symptoms were stable, lenvatinib therapy was continued at 10 mg/day. Over the next few months, the patient experienced a slow but sustained improvement in mobility with an improved ECOG PS of 2, as well as a significant reduction in analgesia use. Her Tg levels also began to decrease (down to 6,660 ng/ml in January 2018; **Table 1**). As treatment continued, the patient regained sensation in her feet and became increasingly able to move independently without the use of walking aids (ECOG PS of 1).

In January 2018, the patient asked to increase the current dose of lenvatinib to 14 mg/day, against the advice of the specialist oncologist on the video-link consultation. The patient felt that her adverse events were under control, with platelet levels maintained above 100/µl, stable liver and renal function, and stable blood pressure; she felt that a higher lenvatinib dose would mean improved control of metastatic disease. However, the increased dose of lenvatinib led to an increase in blood pressure, requiring further changes to antihypertensive medications (dose adjustments and the temporary addition of bisoprolol). With the increase in antihypertensive medications came metabolic disturbance, significant peripheral edema, and a decrease in ECOG PS and quality of life. On the advice of the specialist oncologist, *via* video-link consultation, the lenvatinib dose was reduced back to 10 mg/day. With this change, the patient’s antihypertensive medications were also reduced to previous dose levels, and her condition improved to that reported before the lenvatinib dose increase. Regular imaging has shown neither deterioration of known sites of disease, nor the appearance of new metastatic lesions.

In April 2020, some two and a half years after starting lenvatinib, the patient’s Tg levels have increased steadily, to 20,357 ng/ml, but imaging scans indicate a generally stable condition. Importantly, the patient’s ECOG PS has improved from 3 (at the start of treatment with lenvatinib) to 1, and her symptoms and analgesia requirements are stable. Indeed, the patient has been well enough to attend family and cultural events at many miles from home since 2018, although her mobility level has deteriorated somewhat.

## Discussion

Presentation of metastatic differentiated thyroid cancer as a single sacral metastasis is uncommon (3). As this type of cancer is often amenable to treatment (4), prompt diagnosis is essential to optimize patient outcomes. Although diagnosis in patients in rural areas may be more rapid than in urban populations because of reduced wait times to see a doctor, there are still significant challenges in the treatment and care of a patient with a rare cancer living in a small rural community at a great distance from a specialist cancer center (5–8). The approach to the management and treatment of an elderly person with radioiodine-resistant metastatic thyroid cancer and limited mobility living in a small rural community, as presented in this case report, raises several interesting points for consideration.

Radioiodine-resistant metastatic differentiated thyroid cancer is rare, and disease progression is variable (9). Some patients can have slowly progressive disease, with little or no effect on the patient’s quality of life for some time, while others can exhibit rapid progression and the need to commence systemic treatment (10). This systemic treatment can cause significant toxicity and requires initial frequent monitoring and assessment by a multidisciplinary team familiar with the treatment options (currently [as of April 2020], either lenvatinib or sorafenib) (9, 11). This poses a challenge for health services responsible for small populations spread over a large and rugged geographical area. Similar challenging rural environments for patients with rare cancers requiring specialist oversight occur throughout the world. The patient in this report lived over 200 miles from the specialist thyroid cancer regional center in Aberdeen and travelling from home would require either a flight of 45 minutes, or a 4.5–5-hour drive time each way for at least 16 appointments, a significant investment of time and money and substantial disruption to the patient’s daily life. However, the use of video-conferencing with the specialist oncologist, and most importantly collaboration with not only the local cancer nursing team but also the patient’s general practitioner (GP), enabled successful management of the patient’s adverse events and, ultimately, stabilized the disease.

Although there can be significant challenges with staffing levels in small, remote, rural communities, the staff who work there are typically dedicated and keen to do what is best for their patients. They are used to not having specialist staff close at hand, and are willing to employ a remote, multidisciplinary approach to patient care. For this patient, the team consisted of the specialist based in Aberdeen, the local cancer nursing specialists, the local GP, staff in the local district hospital (for CT scanning), and oncology staff in the regional hospital in Inverness serving the patient’s locality. The cancer nurse specialist (or the deputy when the cancer nurse specialist was unavailable) was the critical link in coordinating the patient’s care and monitoring toxicity. These nurses not only have training in palliative care but also in the treatment of patients with systemic oncology therapies, with some procedures being delivered in the local hospital. This mixture of skills makes cancer nurse specialists well placed to be involved in the care of patients with rarer cancers. Additional training was provided to the nurses by Eisai, the manufacturers of lenvatinib. The success in this case is due to the collaborative approach of a number of clinicians working together with a motivated and well-informed patient. Clear communication among the members of this small team was critical to successful remote management of this patient and might be more difficult with larger teams.

Rural living alters patient access, and perhaps attitudes, to care (5, 6), and the need to travel for treatment may alter decisions to undergo certain therapies. It has been recognized that rural patients tend to have poorer outcomes, and in some cases present to their GP with more advanced disease (6), although this is not necessarily always the case (7). It is important that rural patients do not miss out on newer therapies that could be administered locally, despite the challenges of a remote location, by employing techniques such as coordinated remote care, using video-conferencing for example, with the staff in the specialist centers.

In this case, later presentation may have had an impact on the extent of disease at diagnosis and response to initial treatments. Lenvatinib, as an oral therapy, offered a good opportunity for further treatment of the patient at home, despite the refractory nature of her widespread disease to usual treatments. Coordination of care by the remote cancer specialist *via* the locally based cancer nursing specialist and the patient’s GP, as demonstrated in this case, can reduce the need for patients to travel long distances (12). However, this situation may require further education of the care providers involved with regard to newer treatments to ensure best outcomes (12).

The challenge of managing rare diseases in rural locations is an area of medicine not yet widely researched, with low volumes of evidence being produced. Therefore, this is an area with potential for further research as increasing numbers of new, targeted treatments emerge for patients with cancer who present with more-disseminated and treatment-resistant disease.

## Data Availability Statement

The datasets presented in this article are not readily available because they contain identifying information. Requests to access the datasets should be directed to LS, leslie.samuel@abdn.ac.uk.

## Ethics Statement

Ethical review and approval was not required for the study on human participants in accordance with the local legislation and institutional requirements. The patients/participants provided their written informed consent to participate in this study.

## Author Contributions

RW: idea, writing and review of paper. ML: data gathering and review of paper. MW: data gathering, writing and review of paper. LS: idea, data gathering, writing and review of paper. All authors contributed to the article and approved the submitted version.

## Funding

Medical writing support was provided by Rachel C. Brown, PhD, of Oxford PharmaGenesis Inc., Newtown, PA, USA, and was funded by Eisai Inc., Woodcliff Lake, NJ, USA. The final draft was reviewed by Eisai Inc., Woodcliff Lake, NJ, USA.

## Conflict of Interest

LS: Honorarium and research funding to institution from Eisai.

The remaining authors declare that the research was conducted in the absence of any commercial or financial relationships that could be construed as a potential conflict of interest.

## References

[1] NHS Highland Public Health The Annual Report of the Director of Public Health 2019: Past, Present and Future Trends in Health and Wellbeing (2019). Available at: https://nhshighland.publichealth.scot.nhs.uk/wp-content/uploads/2019/11/DPH-Annual-Report-2019-and-appendices.pdf (Accessed May 8, 2020).

[2] Cramp GJ Development of an integrated and sustainable rural service for people with diabetes in the Scottish Highlands. Rural Remote Health (2006) 6:422. 16475874

[3] Siddiq S Ahmad II Colloby P Papillary thyroid carcinoma presenting as an asymptomatic pelvic bone metastases. J Surg Case Rep (2010) 2010:2. 10.1093/jscr/2010.3.2 PMC3649091 24946172

[4] Khatami F Larijani B Nikfar S Hasanzad M Fendereski K Tavangar SM Personalized treatment options for thyroid cancer: current perspectives. Pharmgenomics Pers Med (2019) 12:235–45. 10.2147/PGPM.S181520 PMC6750856 31571972

[5] Murage P Murchie P Bachmann M Crawford M Jones A Impact of travel time and rurality on presentation and outcomes of symptomatic colorectal cancer: a cross-sectional cohort study in primary care. Br J Gen Pract (2017) 67:e460–6. 10.3399/bjgp17X691349 PMC5565861 28583943

[6] Sabesan S Piliouras P Disparity in cancer survival between urban and rural patients—how can clinicians help reduce it? Rural Remote Health (2009) 9:1146. 19621978

[7] MacVicar E Ritchie D Murchie P Parnaby C MacKay C Ramsay G Analysing the impact of living in a rural setting on the presentation and outcome of colorectal cancer. A prospective single centre observational study. Surgeon (2020) 18(6):354–9. 10.1016/j.surge.2020.02.001 32184069

[8] Baade PD Dasgupta P Aitken J Turrell G Geographic remoteness and risk of advanced colorectal cancer at diagnosis in Queensland: a multilevel study. Br J Cancer (2011) 105:1039–41. 10.1038/bjc.2011.356 PMC3185960 21897391

[9] Nair A Lemery SJ Yang J Marathe A Zhao L Zhao H FDA Approval Summary: Lenvatinib for progressive, radio-iodine-refractory differentiated thyroid cancer. Clin Cancer Res (2015) 21:5205–8. 10.1158/1078-0432.CCR-15-1377 26324740

[10] Kreissl MC Janssen MJR Nagarajah J Current treatment strategies in metastasized differentiated thyroid cancer. J Nucl Med (2019) 60:9–15. 10.2967/jnumed.117.190819 30190306

[11] Cabanillas ME Habra MA Lenvatinib: role in thyroid cancer and other solid tumors. Cancer Treat Rev (2016) 42:47–55. 10.1016/j.ctrv.2015.11.003 26678514

[12] Hall SJ Samuel LM Murchie P Toward shared care for people with cancer: developing the model with patients and GPs. Fam Pract (2011) 28:554–64. 10.1093/fampra/cmr012 21467132

